# Experimental evidence for compositional syntax in bird calls

**DOI:** 10.1038/ncomms10986

**Published:** 2016-03-08

**Authors:** Toshitaka N. Suzuki, David Wheatcroft, Michael Griesser

**Affiliations:** 1 Department of Evolutionary Studies of Biosystems, SOKENDAI (The Graduate University for Advanced Studies), Kamiyamaguchi 1560-35, Hayama, Kanagawa 240-0193, Japan; 2 Department of Life Science, Rikkyo University, Nishi-Ikebukuro 3-34-1, Toshima, Tokyo 171-8501, Japan; 3 Department of Ecology and Genetics, Uppsala University, Norbyvägen 18D, SE-752 36 Uppsala, Sweden; 4 Anthropological Institute and Museum, University of Zurich, Winterthurerstrasse 190, 8057 Zürich, Switzerland

## Abstract

Human language can express limitless meanings from a finite set of words based on combinatorial rules (i.e., compositional syntax). Although animal vocalizations may be comprised of different basic elements (notes), it remains unknown whether compositional syntax has also evolved in animals. Here we report the first experimental evidence for compositional syntax in a wild animal species, the Japanese great tit (*Parus minor*). Tits have over ten different notes in their vocal repertoire and use them either solely or in combination with other notes. Experiments reveal that receivers extract different meanings from ‘ABC' (scan for danger) and ‘D' notes (approach the caller), and a compound meaning from ‘ABC–D' combinations. However, receivers rarely scan and approach when note ordering is artificially reversed (‘D–ABC'). Thus, compositional syntax is not unique to human language but may have evolved independently in animals as one of the basic mechanisms of information transmission.

## Abstract (web summary)

Animal vocalizations contain distinct elements, but it is not clear whether they convey combined meanings in the same way as human speech. Here, Suzuki *et al.* show that Japanese great tits can combine different elements of vocal signals so that they have compositional syntax.

## Graphical abstract

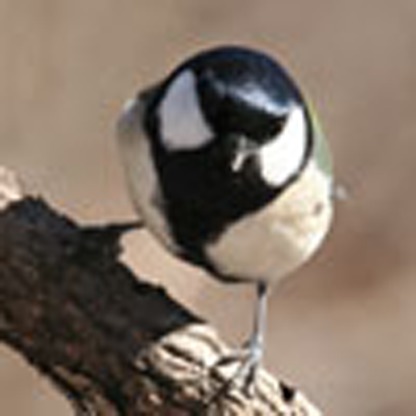

A prominent feature of human language is its combinatorial power, which allows us to generate innumerable expressions from a finite number of vocal elements and meanings^1^,^2^,^3^. Language has two hierarchical levels of syntactic structure: one combines otherwise meaningless elements to form meaningful words (phonology) and the other combines different words to form more complex expressions (compositional syntax)^4^,^5^,^6^. Animal communication systems share many of the basic properties of human language. For example, mammals and birds can use specific call types to denote specific predator categories (i.e., referential communication)^7^,^8^ and can learn to recognize the meaning of calls given by other individuals^9^. Although combinations of discrete vocal elements have been found in some mammals and birds^10^, it remains controversial whether the ability to combine elements is linked to the creation of more complex meanings^6^,^11^.

Recent field studies have suggested that particular combinations of sounds may be linked to particular meanings. For example, white-handed gibbons (*Hylobates lar*) alter the sequence of notes (that is, basic vocal elements) in their vocalizations when informing group members about predatory threats or conspecific intrudors^12^. Similarly, chestnut-crowned babblers (*Pomatostomus ruficeps*) combine two types of notes into two sequences that have different meanings^13^. In both cases, the sounds that constitute the sequence of notes have no apparent communicative meaning on their own and, therefore, these combinations are considered to be phonological^5^,^6^. In contrast, the evidence for compositional syntax remains inconclusive. Campbell's monkeys (*Cercopithecus campbelli*) can modify alarm calls by adding ‘–oo', increasing the generality of the call meaning^14^. However, ‘–oo' is never used alone and, consequently, it is a *suffix* rather than a sound with a distinct meaning^15^. Similarly, putty-nosed monkeys (*Cercopithecus nictitans*) combine discrete alarm calls that denote different predator types to elicit group movements^16^,^17^, but call receivers do not extract a compound meaning from the call combination^18^. Thus, it remains unknown whether animals have evolved compositional syntax or whether this is a unique feature of human language^6^.

Here we provide, to our knowledge, the first unambiguous experimental evidence for compositional syntax in a non-human vocal system. Birds within the family Paridae produce structurally complex vocalizations (‘chicka' or ‘chick-a-dee' calls) that are composed out of different note types (for example, A, B, C and D)^19^. Individuals use these calls in a range of contexts, such as to communicate the discovery of food sources^20^,^21^, when approaching predators to deter them (i.e., mobbing)^22^,^23^,^24^,^25^, or to maintain social cohesion with conspecifics^26^,^27^. Previous studies suggested that different note types have different functions. For example, Carolina chickadees (*Poecile carolinensis*) incorporate a greater number of D notes when discovering a food source or when mobbing a higher-risk predator, and D-rich calls serve to attract flock members to the callers^20^,^23^. These birds incorporate more A notes when discovering an aerial predator^28^ and more C notes when flying^29^. However, because of the lack of playback studies testing the function of individual notes and their combinations, it is still uncertain whether these notes function as different meaningful elements and if these combinations yield a corresponding complexity in call meanings.

In this study, we investigated whether different note types produced by Japanese great tits (*P. minor*; Paridae) have distinct meanings to receivers when produced separately and, if so, whether receivers extract a compound meaning when both elements are combined (compositional syntax). Tits produce ‘chicka' calls when approaching and mobbing predators, and these calls contain a number of unique call types composed of different note types, mainly A, B, C and D notes^25^. A, B and C notes are typically produced in combination with other note types, resulting in AC, BC or ABC calls (Fig. 1a). In contrast, D notes are produced as a string of seven to ten notes (hereafter referred to as a D call, Fig. 1b) and are also used in non-predatory contexts, such as when a bird visits its nest alone and is recruiting its mate (Fig. 2). In predatory contexts, D notes are often produced in combination with other note types and typically appear at the end of note strings, such as AC–D, BC–D or ABC–D calls (Fig. 1c) (ref. 25). Thus, D notes are both produced alone and in combination with other notes, suggesting that they modify the meaning of ABC calls to elicit appropriate mobbing responses to different predator types^25^.

We hypothesized that the combination of ABC calls and D calls into ABC–D calls represents semantically compositional syntax (Fig. 1a–c). To test this hypothesis, we designed two playback experiments. In Experiment 1, we examined whether tits hearing combined ABC–D calls extract the meanings of both ABC and D calls. If tits show a combined response to ABC–D calls, this could be explained by at least two mechanisms. First, tits may combine the distinct behaviour they produce when they hear ABC calls together with the behaviour they produce when they hear D calls, because they recognize ABC–D calls as a single meaningful unit (i.e., compositional syntax). Alternatively, tits may produce the two distinct behavioural responses (that is, first to ABC calls and then to D calls) simply because of the close temporal proximity of ABC and D calls. To differentiate between these two possibilities, we compared the responses of tits with playbacks of natural (ABC–D) and artificially reversed (D–ABC) sequences (Fig. 1d) in Experiment 2. A key predication of the first mechanism is that receivers should produce a compound response only when the combinations of ABC and D calls are produced together according to their note-ordering rule (that is, ABC–D, but not D–ABC). In contrast, according to the second mechanism, receivers should respond similarly whenever ABC and D calls are produced in close proximity, no matter the order in which they are produced.

Here we find that Japanese great tits extract different meanings from ABC and D calls, and a compound meaning from ABC–D calls. As tits fail to produce a compound response when the note sequence is artificially reversed (D–ABC), these findings support the hypothesis that the communication system of tits represents semantically compositional syntax.

## Results

### Experiment 1

Japanese great tits principally displayed two behaviours in response to call playbacks (ABC, D and ABC–D): they scanned the surroundings by turning their heads right and left, and approached the playback loudspeaker. However, they produced these two behaviours differently in response to each of the playback treatments.

During playback of ABC calls, tits continuously turned their heads horizontally on tree branches to scan the surroundings. The rate of horizontal scans varied significantly among the playback treatments; it was higher during playback of ABC calls than during playback of D calls or background noise (control) (generalized linear mixed model: *χ*^2^=62.58, *df*=3, *P*<0.001, Fig. 3a). There were no significant effects of trial order (*χ*^2^=1.14, *df*=1, *P*=0.29) or sex of the focal individuals (*χ*^2^=0.01, *df*=1, *P*=0.92) on the rate of horizontal scans. Pairwise comparisons of treatments showed that the ABC call treatment resulted in significantly more horizontal scans than the D call treatment (Wilcoxon signed-rank tests: *n*=21, *P*<0.0001) and background noise control (*P*<0.001), whereas D calls and background noise were not significantly different (*P*=0.11).

In response to D calls, tits were more likely to approach within 2 m of the playback loudspeaker than in response to ABC calls or background noise. There was a significant effect of playback treatments on the probability of approaching (generalized linear mixed model: *χ*^2^=34.56, *df*=2, *P*<0.001; Fig. 3b), whereas trial order (*χ*^2^=1.47, *df*=1, *P*=0.23) or sex of the focal birds (*χ*^2^=1.93, *df*=1, *P*=0.16) had no significant effects. Pairwise comparisons showed that tits approached the loudspeaker during playback of D calls more often than during playback of ABC calls (sign tests: *P*<0.01) or background noise (*P*<0.01), whereas the responses to ABC calls and background noise were not significantly different (*P*=0.91). These results demonstrate that tits produce distinct behavioural responses when hearing ABC calls (scanning the surroundings) and D calls (approaching the sound source).

In response to playback of ABC–D calls, tits scanned the surroundings more than when hearing D calls (Wilcoxon signed-rank test: *n*=21, *P*<0.001) or background noise (*P*<0.001) and not differently to when hearing ABC calls alone (*P*=0.11; Fig. 3a). However, tits were also more likely to approach within 2 m of the loudspeaker than when hearing ABC calls (sign-tests: *P*=0.02) or the background noise control (*P*<0.01). There was no significant difference in approaching response between ABC–D and D calls (*P*=0.91; Fig. 3b). These results demonstrate that the combined ABC–D calls cause tits produce a combined response containing both behaviours typical of individuals exposed to ABC calls (scanning the horizon) and those typical of individuals exposed to D calls (approaching the sound source).

Across all trials, there was no significant correlation between horizontal scanning and approaching behaviour (Spearman rank-order correlation: horizontal scans versus approaching loudspeaker: *ρ*=0.053, *n*=84, *P*=0.63), indicating that the tits controlled these two behaviours independently.

### Experiment 2

Tits responded differently to playbacks of ABC–D (natural sequence) and D–ABC (artificially reversed sequence) calls. In response to the playback of ABC–D calls, focal birds typically approached within 2 m of the loudspeaker, while scanning the horizon, similar to Experiment 1. However, in response to the playback of D–ABC calls, tits made fewer horizontal scans (generalized linear model: *χ*^2^=27.09, *df*=1, *P*<0.0001; Fig. 4a) and only rarely approached the loudspeaker (*χ*^2^=6.03, *df*=1, *P*=0.014; Fig. 4b). There was no significant difference between sexes in horizontal scans (*χ*^2^=1.05, *df*=1, *P*=0.31) nor approaching behaviour (*χ*^2^=0.002, *df*=1, *P*=0.96). These results demonstrate that tits produce a compound response when ABC and D are combined according to a note-ordering rule, but not when these two note units are simply produced in close temporal proximity.

## Discussion

Our results show that Japanese great tits discriminate between different calls containing different note types: they scan the horizon in response to ABC calls, whereas they approach the sound source in response to D calls. These results indicate that these two calls function as different meaningful units to receivers. ABC calls serve as warning calls that elicit predator-scanning behaviour, whereas D calls serve as recruitment calls that attract conspecifics to the callers. These findings are consistent with previous research showing that A, B and C note combinations are used in response to predators^25^, whereas D notes on its own are used to recruit conspecifics (Fig. 2).

In response to ABC–D calls, Japanese great tits both scan the surroundings and approach the sound source, indicating that they extract the meanings of both ABC and D calls from combined ABC–D calls. In addition, we find no correlation between scanning and approaching behaviours, which enables tits to perform and combine these behaviours flexibly according to the presence and absence of each note unit within calls. Moreover, tits reduce horizontal scanning and rarely approach the loudspeaker when the ordering of the two note units is artificially reversed (D–ABC). These results indicate that the tits perceive ABC–D calls as a single meaningful unit but not as two separated meaningful units (ABC and D calls) simply produced in close proximity. As ABC and D notes convey unique meanings and can be used alone^25^, the combination of these two notes does not meet the criteria of phonology^5^,^6^. In addition, unlike call combinations reported in several non-human primates^14^,^15^,^16^,^17^,^18^, the combination of ABC and D calls conveys a compound meaning that originates from both of the note units. Thus, we conclude that the combination of ABC and D calls in the Japanese great tit obeys semantically compositional syntax^6^.

Previous studies have shown that parids (chickadees and titmice) alter the repetition rate of particular note types (for example, D notes), which elicits different degrees of response in receivers (i.e., graded call system)^22^,^23^,^24^. One explanation for why tits produce different responses to combined ABC–D calls is that D notes increase the salience of ABC calls (or *vice versa*), rather than alter their meaning through a syntactic rule. However, we find no evidence supporting this explanation. In Experiment 1, our data show that tits do not alter the intensity of their responses according to the variation in note repetition rate; they scan with similar intensity to both ABC (3 notes) and ABC–D calls (10–13 notes) and, likewise, approach in response to both D (7–10 notes) and ABC–D calls (10–13 notes). Therefore, neither ABC nor D calls simply modify the intensity of behavioural responses. In addition, using a matched-pairs or balanced design controls for the possibility that any acoustic features other than either note combinations (Experiment 1) or note ordering (Experiment 2) influenced the interpretation of the results (see Methods).

Using a compositional syntax is likely to provide adaptive benefits to Japanese great tits. Similar to many small songbirds, tits face a variety of predatory threats requiring complex behavioural responses^30^,^31^,^32^. Previous studies have demonstrated that avian antipredator communication is adapted to such complexity: some birds produce different calls for different types of threats (for example, different predator types or behaviours) and receivers respond to the calls with appropriate behaviours^30^,^31^,^32^,^33^,^34^,^35^, leading to positive fitness consequences^30^,^32^,^36^. Our results show that the first units of great tits' combinatorial calls (ABC calls) serve as general warning calls, whereas the last units (D calls) serve as recruitment calls. The specific combination of these calls may serve as an adaption to facing predators that require complex behaviours to be effectively detected and monitored. For example, scanning the surroundings is likely to allow a tit to efficiently detect a flying predator, such as a crow that can approach a nest from all directions^31^. In contrast, predators that only approach the nest from below, such as martens, are likely to be effectively detected and monitored both by approaching the caller and scanning the surroundings. Japanese great tits incorporate a greater number of D notes into other note units, such as ABC, when mobbing martens than when mobbing crows^25^. This suggests that tits have co-opted the signal normally used to recruit other individuals (for example, to coordinate parental feeding visits), to stimulate receivers to perform an appropriate combination of behaviours.

In addition, we suggest that the specific note-ordering rule (ABC calls before D calls) used by Japanese great tits in anti-predator contexts may be an adaptation to the greater importance of effectively and quickly warning conspecifics about the presence of predators before transmitting any additional behavioural cues. As D notes are often produced in non-predator contexts, conspecifics hearing D notes before ABC notes may be slower to produce appropriate anti-predator behaviours, which may be of particular importance when tits are defending their nestlings^25^,^30^.

Although we provide evidence for compositional syntax in the combination of ABC and D calls, it is not yet clear how the meaning of ABC calls is generated. One possibility is that A, B and C notes have different meanings and their combination has a compound meaning (i.e., compositional syntax). However, these notes may be meaningless as their own, but the combinations make the meaningful units that elicit scanning behaviour in receivers (i.e., phonology). Support for this idea comes from the observation that tits use A, B and C notes in many different combinations (for example, AB, AC and BC) when mobbing predators^25^. Therefore, it might be possible that all these combinations potentially encode the same threat information; however, the difference in note combinations or sequences of different call types may encode additional information, such as individual identity of callers. Note combinations are widely documented in other members of the Paridae, but their complexity may differ across species^37^. Further comparative studies may provide insight into the socio-ecological factors^38^ that drive the evolution of combinatorial signalling such as phonology and compositional syntax.

In conclusion, we provide the first experimental evidence for compositional syntax in a non-human vocal system. Over the past decades, many key attributes of human language have been reported from animal species: vocal learning^9^,^39^, referential communication^7^,^8^ and phonology^12^,^13^. Our results extend these studies and challenge the long-standing view that compositional syntax is unique to human language^5^,^6^. Although previous studies on syntactic communication mainly focused on primates^12^,^14^,^15^,^16^,^17^,^18^, our findings highlight that the ability to recognize the combinations of different meaningful units as compositional calls has evolved in birds. Signal combinations can increase the number of meanings that individuals can convey from a limited number of vocal elements and provide the basis for the generation of novel signals. Uncovering the cognitive mechanisms and socio-ecological functions of syntactic communication in animal models may provide insights into the evolution of structural complexity of human language.

## Methods

### General experimental design

This study consisted of two playback experiments. Experiment 1 was designed to test whether Japanese great tits discriminate between calls with different note types (ABC and D calls) and, if so, whether they also extract a compound meaning from combined calls (ABC–D). If the combination of ABC and D calls obeys compositional syntax, tits are expected to show different responses to the two different note units and a compound response to the combined calls. We examined the response of Japanese great tits to playbacks of ABC calls, D calls, ABC–D calls and the background noise (control).

Experiment 2 was designed to test whether tits respond to the combination of ABC and D calls through the recognition of the note-ordering rule. If they perceive the combined calls (ABC–D calls) as a single meaningful unit but not as separated and independent calls (ABC and D calls), they are expected to respond differently to the natural (ABC–D) and reversed (D–ABC) sequences. We tested the response of tits to playbacks of ABC–D and D–ABC calls.

### Study population and call recordings

Experiments were conducted in a colour-ringed population of Japanese great tits in a mixed deciduous–coniferous forest near Karuizawa, Nagano Prefecture, Japan (36°19′–22′N, 138°32′–37′E). For all playbacks, we used ‘chicka' mobbing calls that were previously recorded from Japanese great tits (ten males and seven females) from the study population in 2009 and 2010 (refs 25, 30). The ‘chicka' calls were elicited by exposure to either a taxidermic model of a crow or a marten near the nest boxes. Calls were recorded using an LS370 parabolic microphone (Fuji Planning Corporation, Tokyo, Japan) connected to an R-09HR digital audio recorder (sampling rate, 48 kHz; sample size, 16 bits; Roland Corporation, Shizuoka, Japan). Detailed information on call recordings has been provided elsewhere^25^,^30^.

### Playback stimuli

Adobe Audition 3.0 software and Raven Pro 1.3 software^40^ were used to construct the playback stimuli. We chose four types of notes (A, B, C and D) from recordings of every source individual on the basis of the sound quality (for example, the bird was close to the microphone when it called and the background noise was low). Although A, B and C notes were typically produced as a single note in a call, D notes always occurred as a string of multiple notes. Therefore, we used a single A, B and C note and a string of seven to ten D notes to construct the playback calls. These four note types were combined into an ABC–D call with natural intervals between the notes (50–150 ms, measured for each individual of the recording source). We thus obtained a total of 21 ABC–D calls from the recording files (11 calls from the recordings of 10 males and 10 calls from the recordings of 7 females).

**Figure 1:**
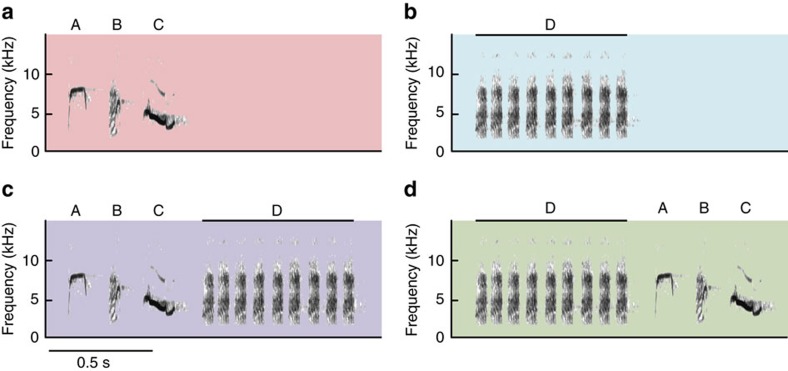
Sound spectrograms of call treatments played to Japanese great tits. (**a**) ABC call is composed of single A, B and C notes. (**b**) D call is composed of seven to ten D notes. (**c**) ABC–D call is the combination of ABC and D calls. (**d**) D–ABC call is a reversed combination of ABC and D calls. These calls were digitally edited using Raven Pro 1.3 software.

**Figure 2:**
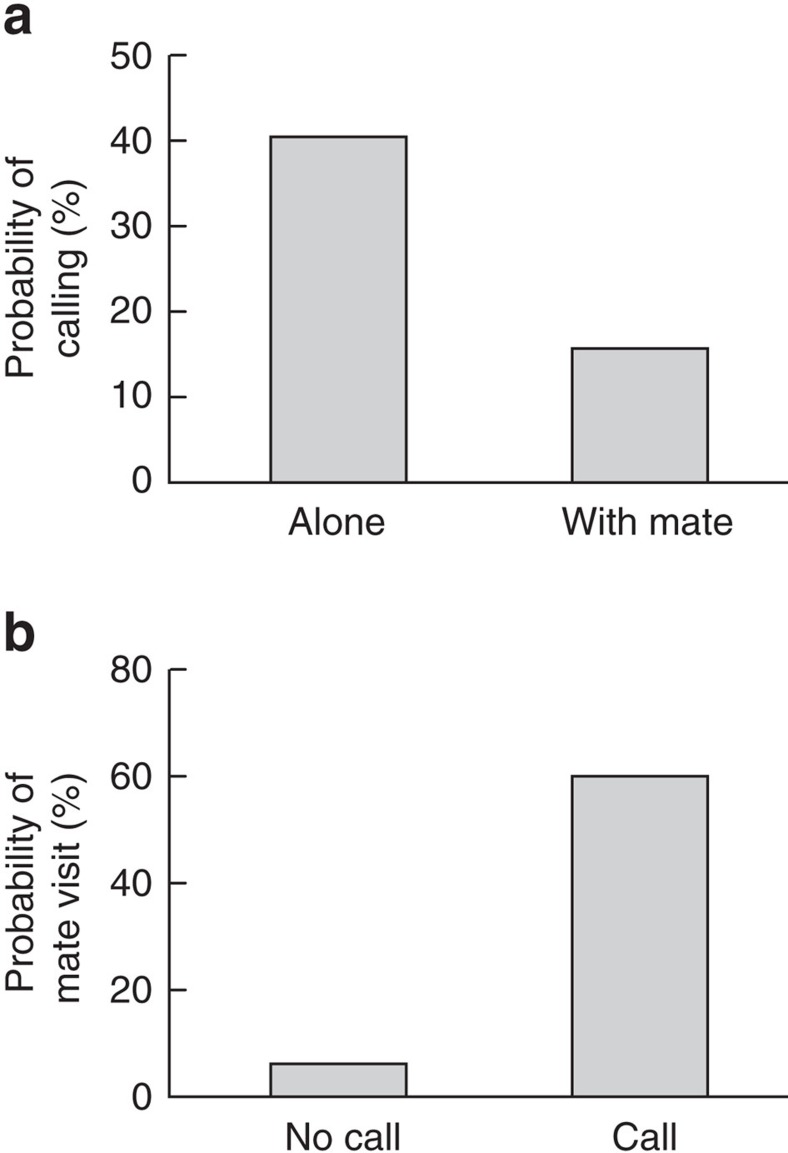
Usage of D calls in a non-predatory context in Japanese great tits. (**a**) Effect of the presence of a mate on the production of D calls (*n*=187 observations, *n*=40 individuals): tits produced D calls more often when they visited the nest alone than when they did following their mate (generalized linear mixed model: *χ*^2^=5.00, *df*=1, *P*=0.025), after controlling for the nonsignificant influence of sex of the callers (*χ*^2^=1.16, *df*=1, *P*=0.281). (**b**) Effect of D calls on the recruitment of their mate (*n*=136 observations, *n*=34 individuals): tits that produced D calls were more likely to subsequently attract their mates than tits that did not produce D calls (generalized linear mixed model: *χ*^2^=35.37, *df*=1, *P*<0.0001), even after controlling for a significant influence of the responding mate's sex (males were more likely to approach D calls given by their partners than were females; *χ*^2^=9.32, *df*=1, *P*=0.002).

**Figure 3:**
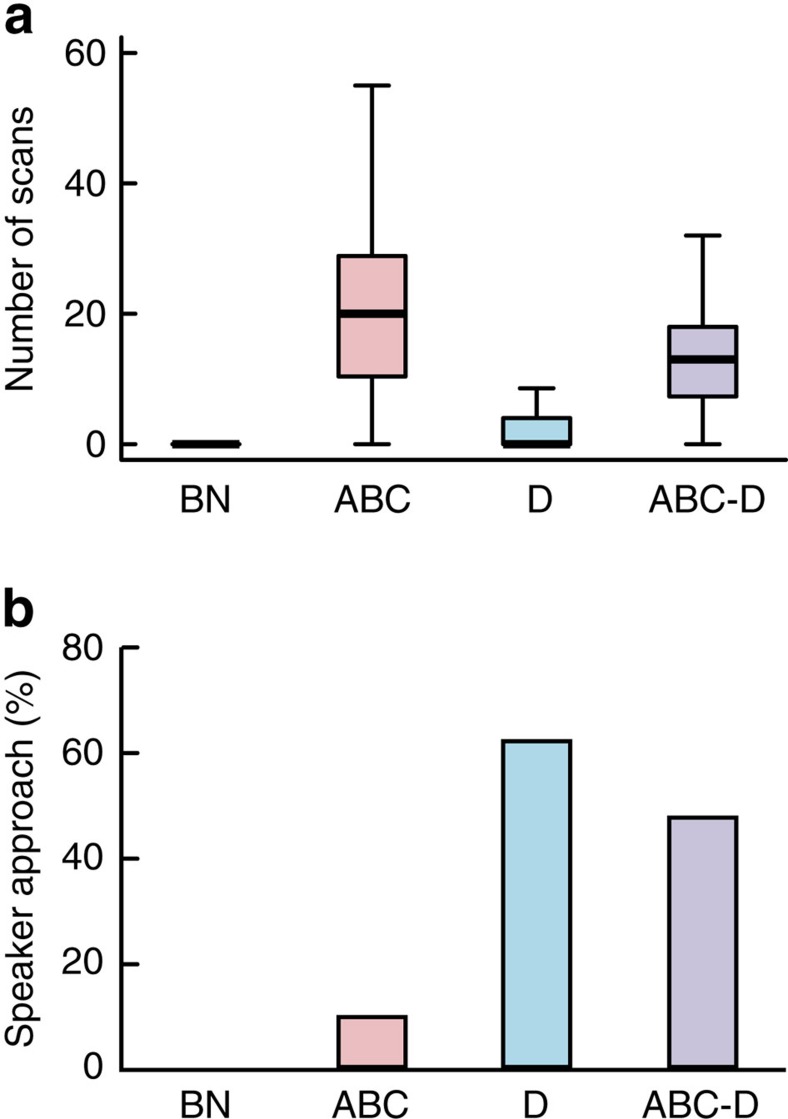
Responses of Japanese great tits to playbacks of ABC, D and ABC–D calls, and background noise (BN). (**a**) Number of horizontal scans made by tits in 90 s (generalized linear mixed model: *χ*^2^=62.58, *df*=3, *P*<0.001). (**b**) Percentage of trials in which tits approached within 2 m of the loudspeaker (generalized linear mixed model: *χ*^2^=34.56, *df*=2, *P*<0.001). The box and whisker plots display the median value and 25 and 75% quartiles; the whiskers are extended to the most extreme value inside the 1.5-fold interquartile range. Sample size: *n*=21 individuals. Each individual was exposed to all four treatments in varied orders, giving *n*=21 samples per treatment.

**Figure 4:**
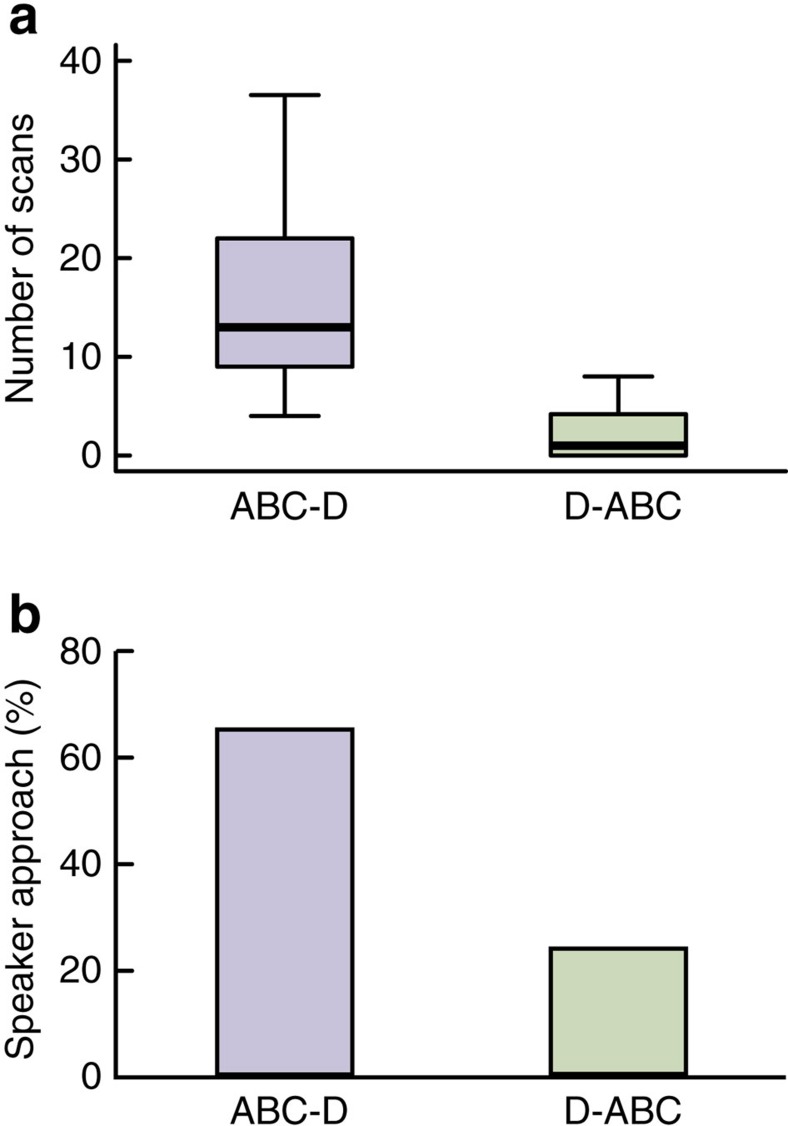
Responses of Japanese great tits to playbacks of ABC–D and D–ABC calls. (**a**) Number of horizontal scans made by tits in 90 s (generalized linear model: *χ*^2^=27.09, *df*=1, *P*<0.0001). (**b**) Percentage of trials in which tits approached within 2 m of the loudspeaker (generalized linear model: *χ*^2^=6.03, *df*=1, *P*=0.014). The box and whisker plots display the median value and 25 and 75% quartiles; the whiskers are extended to the most extreme value inside the 1.5-fold interquartile range. Sample size: *n*=34 individuals. Each individual was exposed to only one treatment, giving *n*=17 samples per treatment.

In Experiment 1, we prepared three call treatments (ABC, D and ABC–D calls; Fig. 1a–c) and a control treatment (background noise). ABC and D call types were constructed by eliminating either D or ABC note units from each of the 21 ABC–D calls. Calls were repeated in a sound file at a rate of 30 calls per minute (one call every 2 s, total duration 90 s). This calling rate is within the range of the natural repetition rates for ‘chicka' calls during the nestling period^25^,^30^. Low-frequency noise (<1 kHz) was filtered out and the calls were amplified on a computer. The background noise files were created in the same way as the call files, using the parts where no birds were calling in the same recordings as call treatments. Thus, we constructed 21 unique sets of playback stimuli (ABC, D and ABC–D calls, and background noise). To avoid pseudoreplication^41^, we played back each exemplar only once to each focal individual (*n*=21). To each focal individual, we played back three call types that originated from the same calling individual (matched-pairs design), ensuring that any acoustic features other than the note combinations (for example, the intervals between different notes) were constant over these three call treatments. All of the sound files were saved in WAV format (16-bit accuracy, 48.0-kHz sampling rate) onto an SD memory card.

In Experiment 2, we prepared two types of calls: ABC–D (natural sequence) and D–ABC (artificially reversed sequence) calls (Fig. 1d). We chose 17 different ABC–D calls that originated from different individuals (10 male calls and 7 female calls). D–ABC calls (*n*=17) were constructed by using these ABC–D calls and re-ordering the sequence by moving D notes before A notes. The intervals between D and A notes within D–ABC calls were set at the same durations as those between C and D notes in their original ABC–D calls, ensuring that any acoustic features other than note orderings did not differ between ABC–D and D–ABC calls (balanced design). These calls were recorded in a sound file at a rate of 20 calls per minute (one call every 3 s, total duration 90 s), which was saved in WAV format (16-bit accuracy, 48.0-kHz sampling rate) onto an SD memory card. This calling rate is within the natural range^25^,^30^ and ensures that each call is separated by at least 1.6 s from any preceding calls, reducing the chances that receivers could perceive ABC–D sequences from adjacent D–ABC calls. As with Experiment 1, unique exemplars were used for each focal individual to avoid pseudoreplication^41^.

### Experiment 1

We tested the responses of Japanese great tits to playbacks of ABC, D and ABC–D calls. We conducted this experiment on 21 adult great tits (10 males and 11 females from 21 different pairs) during their first breeding attempt of the season. All experimental birds bred in nest boxes that were attached to tree trunks 1.8 m above the ground. The average brood size of these pairs was 7.8±1.5 (mean±s.d., *n*=21). The experimental trials were carried out from 3 June to 15 June 2012 when the nestlings were 10–17 (12.4±1.7) days old.

An AT-SPG50 loudspeaker (Audio-Technica Corporation, Tokyo, Japan) was hung from a tree and fixed 1.8±0.2 m from the ground and 5.3±1.0 m from the nest (mean±s.d., *n*=21). The loudspeaker was connected to an R-09 HR digital audio recorder with EXC-12A extension cords (JVC Kenwood Corporation, Kanagawa, Japan), which enabled the control of playbacks from an observation position 15 m away from the nest. Playbacks commenced when a focal individual was within 5 m of the nest and their mate was absent. Calls were played back at a standardized volume (75 dB re 20 μPa at 1 m from the loudspeaker measured using an SM-325 sound level meter; AS ONE Corporation, Osaka, Japan) and background noise was played back at the same amplitude as the background noise level of the call playbacks (50 dB re 20 μPa at 1 m). Focal birds received playbacks of calls that were constructed from unfamiliar individuals (that is, not their mates or neighbours), to eliminate any influence of familiarity. No more than two trials were conducted at the same nest in a single day and playbacks at the same nest were separated by at least 2 h to reduce habituation. The order of the playbacks was randomized. We used the same position for setting the loudspeaker in all treatments at each site to control for its possible effect on the behavioural response. Trials were conducted in calm and dry weather between 08:30 and 16:00 h (Japan Standard Time).

To determine the tits' responses to different treatments, we recorded the following behavioural variables during 90 s of playbacks: (1) number of horizontal scans: we counted the number of movements that birds made with their heads from left to right or right to left (approximately a 180° turn) and (2) approaching the loudspeaker: we recorded whether birds approached within 2 m of the loudspeaker during the playback. These behavioural variables were commented onto an R-09HR digital audio recorder. We also recorded the latency to feed nestlings by using a GZ-MG880 digital video camera (JVC Kenwood Corporation) set *ca*. 10 m from the nest. Behavioural observations were continued until each playback had ended and the adults entered the nest box to feed the chicks.

### Experiment 2

We tested the responses of Japanese great tits to naturally combined ABC–D calls and artificially reversed D–ABC calls. We conducted this experiment with 34 individual great tits (ABC–D calls: 11 males and 6 females; D–ABC calls: 12 males and 5 females). The minimum distance between experimental sites was 400 m, to ensure the collection of data from different individual tits^21^. Trials were carried out between 6 November and 19 November 2015, during the non-breeding season, when tits, such as other members of the Paridae, are threatened by a variety of predators and produce a corresponding variety of alarm calls^22^,^23^,^24^.

First, we searched for a flock of Japanese great tits. On finding a flock, we hung an AT-SPG50 loudspeaker from a tree at 1.8±0.1 m from the ground (mean±s.d., *n*=34). The loudspeaker was connected to an R-09 HR digital audio recorder with EXC-12A extension cords, which enabled the control of playbacks from an observation position *ca*. 10 m away from the loudspeaker. Then, we commenced the playback when a tit came within 15 m of the loudspeaker. We defined the individual that was closest to the loudspeaker as the focal individual and focussed on this individual during the playback. Trials were carried out under calm and dry weather between 08:45 and 15:30 h (Japan Standard Time). ABC–D and D–ABC treatments were alternated with each other on successive trials so that responses to both treatments were observed under largely similar conditions.

As with Experiment 1, we measured two behavioural variables: (1) number of horizontal scans and (2) the probability of approaching within 2 m of the loudspeaker. These variables were commented onto an R-09HR digital audio recorder.

### Usage of D calls in a non-predatory context

Japanese great tits produce D calls not only in predatory contexts but also in non-predatory contexts such as when visiting their nests. We investigated the usage and function of D calls in a non-predatory context, testing the hypothesis that D calls serve to recruit conspecifics. If this hypothesis is true, then we predict that (1) tits produce D calls more often when they visit the nest alone than when their mated partner is also present and (2) a caller's mate is more likely to visit the nest when the caller produces D calls than when it does not. We therefore investigated the effect of social context on the usage of D calls and whether the production of D calls increases the visitation of their mate to the nest.

We observed *n*=187 nest visitations of 40 adults (19 males and 21 females) at 22 nests from 3 June to 15 June 2012, when nestlings were 10–17 days old. When a parent visited within 5 m of the nest box with a food item, we noted (1) the sex of the parent, (2) whether it gave D calls and (3) whether its mate was present within 5 m of the nest box. In the case in which a parent visited the nest alone (*n*=136), we also noted (4) whether the mate visited within 5 m of the nest before the first bird entered the nest box. Observations were made at 15 m from the nest box, a distance from which the tits' behaviour was not disturbed.

### Statistical analysis

All the statistical analyses were performed using R for Mac OS X version 3.1.1 (ref. 42). In the analysis of Experiment 1, we used generalized linear mixed models for primary analyses, which include the treatment as a fixed term and individual identity of focal birds as a random term. Trial order and sex were also entered as covariates. We used a negative binomial error distribution and log-link function (*glmer.nb* in the package *lme4* (ref. 43)) for the analysis of the number of horizontal scans and a binomial error distribution and logit-link function (*glmer* in the package *lme4* (ref. 43)) for the analysis of the probability of approaching behaviour (yes or no). In some trials, tits visited the nest boxes and flew out of sight immediately after feeding chicks. Therefore, we determined the time duration in which we could observe the behaviour of the tits as the observation time and included this term in the analysis of horizontal scans as a log-transformed offset. For the analysis of approaching behaviour, it was not possible to run the model because of the absence of variance in background noise control treatment (no birds approached to the loudspeaker during this treatment). Therefore, we combined background noise and ABC calls in this analysis, as there was no significant difference between these two treatments (sign test, *P*=0.5). We used likelihood ratio tests to calculate *P*-values of each term. In the event of a significant effect of treatment, we further conducted pair-wise comparisons by using non-parametric statistics: Wilcoxon signed-ranks tests (*wilcox.paired.multcomp* in the package *RVAideMemoire*^44^) for the number of horizontal scans (standardized by observation time) and sign tests for approaching to the loudspeaker (*cochran.qtest* in the package *RVAideMemoire*^44^). When making these multiple comparisons, sequential Bonferroni corrections were applied for the adjustments of *P*-values. To investigate the correlation between scanning and approaching behaviours, we used Spearman's rank-order correlations (*cor.test* in the default package *stats*).

In the analysis of Experiment 2, we ran generalized linear models including treatment as a fixed term and sex as a covariate. We used a negative binomial error distribution and log-link function (*glm.nb* in the package *MASS*^45^) for the analysis of horizontal scans and a binomial error distribution and logit-link function (*glm* in the package *stats*) for the analysis of approaching behaviour. We standardized the number of scans by observation time, as in some cases the focal individuals flew away from the sight during the trials.

In the analysis of the usage of D calls, we ran generalized linear mixed models with a binomial error distribution and a logit-link function (*glmer* in the package *lme4* (ref. 43)). To test the effect of social context on the production of D calls, we fitted social context (mate present or absent) as a fixed term and the probability of D calling (yes or no) as a dependent variable. To test the effect of D calling on the recruitment of a mate to the nest, we fitted the production of D calls (yes or no) as a fixed term and the probability of recruitment (yes or no) as a dependent variable. In both models, we also included sex of focal birds as a covariate and individual identity of focal birds and individual nest as random terms. All tests were two-tailed and the significance level was set at *α*=0.05.

### Ethical statement

All experiments were performed in accordance with relevant guidelines and regulations. All experimental protocols were approved by the Animal Care and Use Committees at the Rikkyo University and SOKENDAI (The Graduate University for Advanced Studies), and adhered to the Guidelines for the Use of Animals in Research of the Animal Behavior Society/Association for the Study of Animal Behaviour. This research was performed under permission from the Ministry of the Environment and the Forestry Agency of Japan.

## Additional information

**How to cite this article:** Suzuki, T. N. *et al.* Experimental evidence for compositional syntax in bird calls. *Nat. Commun.* 7:10986 doi: 10.1038/ncomms10986 (2016).
